# Utilisation of primary care electronic patient records for identification and targeted invitation of individuals to a lung cancer screening programme

**DOI:** 10.1016/j.lungcan.2022.09.009

**Published:** 2022-11

**Authors:** Jennifer L. Dickson, Helen Hall, Carolyn Horst, Sophie Tisi, Priyam Verghese, Sarah Worboys, Andrew Perugia, James Rusius, Anne-Marie Mullin, Jonathan Teague, Laura Farrelly, Vicky Bowyer, Kylie Gyertson, Fanta Bojang, Claire Levermore, Tania Anastasiadis, John McCabe, Anand Devaraj, Arjun Nair, Neal Navani, Allan Hackshaw, Samantha L. Quaife, Sam M. Janes, SUMMIT consortium

**Affiliations:** a Lungs for Living Research Centre, UCL Respiratory, University College London, London, UK; b James Wigg GP Practice, London, UK; c NOCLOR Research Support, London, UK; d Cancer Research UK and UCL Cancer Trials Centre, University College London, London, UK; e University College London Hospitals NHS Foundation Trust, London, UK; f Tower Hamlets Clinical Commissioning Group, London, UK; g Department of Radiology, Royal Brompton Hospital, London, UK; h National Heart and Lung Institute, Imperial College, London, UK; i Wolfson Institute of Population Health, Barts and The London School of Medicine and Dentistry, Queen Mary University of London, London, UK

**Keywords:** Lung cancer, Lung cancer screening

## Abstract

Lung cancer screening (LCS) eligibility is largely determined by tobacco consumption. Primary care smoking data could guide LCS invitation and eligibility assessment. We present observational data from the SUMMIT Study, where individual self-reported smoking status was concordant with primary care records in 75.3%. However, 10.3% demonstrated inconsistencies between historic and most recent smoking status documentation. Quantified tobacco consumption was frequently missing, precluding direct LCS eligibility assessment. Primary care recorded “ever-smoker” status, encompassing both recent and historic documentation, can be used to target LCS invitation. Identifying those with missing or erroneous “never-smoker” smoking status is crucial for equitable invitation to LCS.

## Highlights

• Primary care records can identify individuals to invite for lung cancer screening risk assessment.
• Invitation eligibility should use current and historic smoking status recording.
• Direct eligibility assessment from primary care data is impracticable with some demographic bias.
• LCS programmes require provision for individual-level eligibility assessment.
• Work is needed to identify those without smoking status in primary care records.

## 1 Introduction

Lung Cancer Screening (LCS) using Low-Dose Computed Tomography (LDCT) reduces lung cancer-specific mortality in high-risk individuals [1], [2]. Unlike other cancer screening programmes for which eligibility is largely based on age and sex (e.g., Breast and Cervical screening), eligibility for LCS is based on the presence of lung cancer risk factors, the two main ones being increasing age and history of tobacco smoking. In the US, eligibility for LCS is therefore based on age and smoking history alone. However, analysis of data from the National Lung Screening Trial (NLST) has demonstrated that LCS is more efficient and cost-effective when using multi-factor individual lung cancer risk calculations which include smoking history [3]. In the UK, no comprehensive system currently exists for assessing smoking history to guide LCS invitation at a population level. However, primary care electronic patient records provide a potential data source for this. While several UK studies have utilised primary care records to target LCS invitation [4], [5], [6], [7], none have reported the accuracy of data used. Previous reports found smoking status recording in primary care to be incomplete and subject to inaccuracies [8], with limited improvements despite incentivisation [9]. A recent evaluation using routinely collected primary care registry data to calculate validated lung cancer risk scores demonstrated a negative impact on model accuracy, with limitations in quality and completeness of data cited as potential contributary factors [10].

This manuscript assesses the completeness and validity of tobacco smoking exposure data extracted from primary care records, to examine whether this could be recommended as a comprehensive method for identifying individuals to invite for LCS.

## 2 Methods

**Fig. 1:**
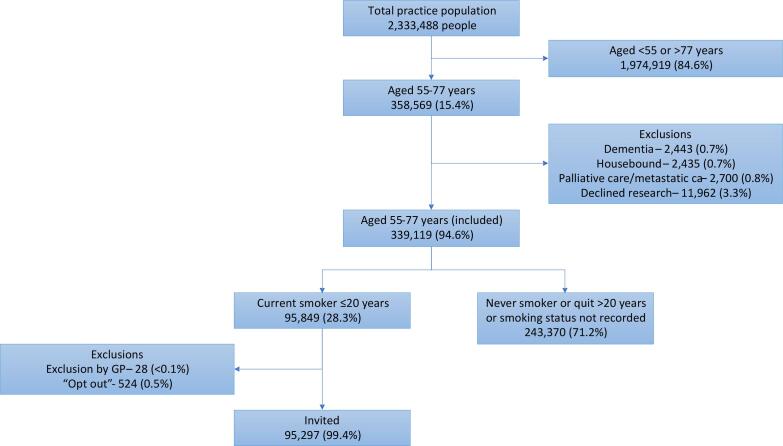
Identification of individuals to invite for a LHC as part of the SUMMIT Study.

The SUMMIT Study is a prospective observational cohort study aiming to assess the implementation of LDCT for LCS in a high-risk population and to validate a multi-cancer early detection blood test. Between March 2019 and December 2019, standardised electronic database searches at participating primary care practices across north central and east London identified individuals for invitation. Criteria for invitation included being aged 55–77 years with a documented status of “current smoker” within the prior 20 years. Individuals on a dementia or palliative care register, that had metastatic cancer, were housebound or had documented refusal to participate in research were excluded (Fig. 1).

Individuals identified as potentially eligible were invited by letter, where if interested they were advised to contact the team via telephone to arrange a Lung Health Check (LHC) appointment. During this telephone call their lung cancer risk was estimated to determine their eligibility for a LHC appointment [11]. At the in person LHC appointment individuals meeting either one of United States Preventive Services Task Force (USPSTF) 2014 criteria [12] or Prostate, Lung, Colorectal, Ovarian (PLCO)_m2012_ 6-year lung cancer risk [13] ≥ 1.3 % were offered LCS. Selection criteria were chosen to closely align with the USPSTF guidelines at the time of study set up. Some criteria were broadened to maximise the inclusion of those potentially eligible.

We analysed the quality of primary care smoking history data, including the proportion of records with missing or inconsistent data, the time since last updated and, for individuals who completed a LCS eligibility assessment, rates of concordance against self-reported data. Associations with sociodemographic factors were examined using logistic regression.

## 3 Results

### 3.1 Completeness and recency of smoking status and tobacco consumption records

Between 20th March 2019 and 12th December 2019, 95,297 individuals from 251 practices were identified as potentially eligible and sent invitation letters (Fig. 1).

Of those invited, 83.8 % (n = 79,826) had their smoking status recorded within the past three years, but a small minority (0.2 %, n = 153) last had this updated > 15 years prior. Amongst current smokers (n = 48,518), tobacco consumption units (i.e., if an individual smoked pre-rolled cigarettes “cigarettes per day” or hand rolled tobacco “grams of tobacco per week”) and quantified measures of consumption (i.e., the average number of cigarettes smoked per day) were recorded in their most recent smoking record in 59.7 % (n = 28,942) and 60.1 % (n = 29,143) respectively. Odds of missing data were highest amongst individuals from less deprived Index of Multiple Deprivation (IMD) quintiles (vs the most deprived quintile) and lower amongst those aged > 70 vs < 55 years (aOR:0.89; 95 % CI:0.81–0.99). The absolute proportion with missing data varied by ethnic group (range: 18.8–47.4 %), with a statistically significant lower likelihood of missing consumption data among individuals of Bangladeshi ethnicity (aOR:0.34; 030–0.38) and higher likelihood among those of mixed white and black Caribbean ethnicity (aOR:1.30; 1.06–1.59), when compared with those of a white British ethnicity (Table 1).

**Table 1:** Frequency and independent predictors of missing b or inconsistent smoking data in primary care records^c^

	Missing tobacco consumption units^b^ in primary care data (all invited current smokers, n=48,518)			Inconsistent smoking status values^c^ in primary care data (all invited, n=95,297)		
	Affected 19,576 (40.3%)	Unadjusted OR (95% CI)	Adjusted OR (95% CI)	Affected 9,826 (10.3%)	Unadjusted OR (95% CI)	Adjusted OR (95% CI)
Sex						
Female	7,827 (40.8)	1.0	1.0	5,232 (13.2)	1	1
Male	11,749 (40.0)	0.97 (0.93 – 1.00) p=0.085	0.9 (0.95 – 1.03) p=0.521	4,594 (8.3)	0.60 (0.57 – 0.62) p<0.001	0.45 (0.43 – 0.47) p<0.001
Missing^a^	0	-	-	1	-	-

Age groups						
55-59	8,070 (40.7)	1.0	1.0	3,216 (9.2)	1	1
60-64	5,397 (40.5)	0.99 (0.95 – 1.04) p=0.779	0.99 (0.95 – 1.04) p=0.722	2,456 (9.8)	1.07 (1.01 – 1.13) p=0.026	1.08 (1.02 – 1.14) p=0.013
65-69	3,305 (40.5)	0.99 (0.94 – 1.04) p=0.702	0.98 (0.92 – 1.03) p=0.375	2,020 (11.5)	1.28 (1.20 - 1.36) p<0.001	1.38 (1.29 – 1.47) p<0.001
70-75	2,079 (39.1)	0.94 (0.88 – 1.00) p=0.038	0.90 (0.84 – 0.96) p=0.001	1,487 (11.6)	1.30 (1.21 – 1.38) p<0.001	1.63 (1.51 – 1.75) p<0.001
>75	718 (38.2)	0.90 (0.82 – 0.99) p=0.033	0.89 (0.81 - 0.99) p=0.03	646 (13.0)	1.47 (1.34 – 1.60) p<0.001	1.74 (1.58 – 1.92) p<0.001
Missing	15	-	-	28	-	-

Ethnicity						
White						
British/mixed British	8,616 (41.0)	1.0	1.0	2,834 (6.7)	1	1
Irish	557 (39.0)	0.92 (0.82 – 1.03) p=0.138	0.93 (0.83 – 1.04) p=0.175	141 (4.9)	0.73 (0.61 – 0.86) p<0.001	0.73 (0.61 – 0.87) p<0.001
Other White	3,424 (41.2)	(0.96 – 1.06) p=0.731	(0.95 – 1.05) p=0.955	1,181 (8.1)	1.24 (1.16 – 1.33) p<0.001	1.34 (1.25 – 1.44) p<0.001

Asian or Asian British						
Indian or British Indian	542 (42.1)	1.05 (0.82 – 1.26) p=0.420	1.03 (0.92 – 1.16) p=0.612	702 (22.9)	4.15 (3.78 – 4.55) p<0.001	5.86 (5.30 – 6.46) p<0.001
Pakistani or British Pakistani	305 (37.9)	0.88 (0.76 – 1.02) p=0.086	0.88 (0.76 – 1.02) p=0.084	374 (21.6)	3.86 (3.42 – 4.35) p<0.001	5.85 (5.16 – 6.63) p<0.001
Bangladeshi or British Bangladeshi	365 (18.8)	0.33 (0.29 – 0.37) p<0.001	0.34 (0.30 – 0.38) p<0.001	1623 (33.8)	7.16 (6.67 – 7.69) p<0.001	9.79 (9.07 – 10.57) p<0.001
Other Asian	393 (40.1)	0.96 (0.85 – 1.10) p=0.57	0.97 (0.85 – 1.10) p=0.623	338 (16.2)	2.70 (2.39 – 3.05) p<0.001	3.46 (3.05 – 3.93) p<0.001

Black						
Caribbean	1,028 (41.8)	1.04 (0.95 – 1.13) p=0.428	1.05 (0.96 – 1.15) p=0.258	516 (11.6)	1.84 (1.67 – 2.03) p<0.001	2.23 (2.02 – 2.48) p<0.001
African	440 (41.0)	1.00 (0.88 – 1.13) p=0.997)	1.02 (0.90 – 1.15) p=0.258	571 (22.2)	4.00 (3.62 – 4.43) p<0.001	5.60 (5.04 – 6.22) p<0.001
Other black	434 (38.5)	0.90 (0.80 – 1.02) p=0.106	1.02 (0.90 – 1.15) p=0.802	207 (10.5)	1.64 (1.41 – 1.90) p<0.001	2.14 (1.85 – 2.50) p<0.001

Mixed						
White and black Caribbean	182 (47.4)	1.30 (1.06 – 1.60) p=0.011	1.30 (1.06 – 1.59) p=0.011	61 (9.5)	1.48 (1.13 – 1.93) p=0.004	1.69 (1.29 – 2.21) p<0.001
White and black African	66 (38.8)	0.91 (0.67 – 1.25) p=0.570	0.93 (0.68 – 1.27) p=0.647	47 (15.0)	2.47 (1.81 – 3.38) p<0.001	3.13 (2.27 – 4.30) p<0.001
White and Asian	80 (44.9)	1.18 (0.87 – 1.58) p=0.284	1.12 (0.83 – 1.51) p=0.462	33 (10.2)	1.60 (1.11 – 2.23) p=0.011	1.79 (1.23 – 2.59) p=0.002
Mixed – other	148 (41.3)	1.02 (0.82 – 1.26) p=0.889	1.03 (0.83 – 1.27) p=0.797	64 (9.2)	1.42 (1.09 – 1.84) p=0.009	1.56 (1.19 – 2.03) p=0.001

Other						
Chinese	122 (36.7)	0.84 (0.67 – 1.05) p=0.121)	0.91 (0.80 – 1.03) p=0.129	79 (11.9)	1.89 (1.49 – 2.40) p<0.001	2.32 (1.82 – 2.96) p<0.001
Other	919 (41.5)	1.02 (0.94 – 1.12) p=0.627	0.81 (0.65 – 1.02) p=0.074	459 (11.0)	1.74 (1.57 – 1.93) p<0.001	2.00 (1.79 – 2.22) p<0.001
Not stated	343 (42.9)	1.08 (0.94 – 1.25) p=0.283	1.02 (0.93 – 1.11) p=0.725	153 (9.8)	1.53 (1.29 – 1.81) p<0.001	1.63 (1.37 – 1.94) p<0.001

Missing	3,638	-	-	6,387	-	-

National Index of Multiple Deprivation						
Quintile 1 (most deprived)	7,453 (38.5)	1	1	3,753 (10.6)	1	1
Quintile 2	6,097 (40.8)	1.10 (1.06 – 1.15) p<0.001	1.08 (1.04 – 1.13) p=0.001	3,003 (10.4)	0.97 (0.93 – 1.02) p=0.29	1.06 (1.01 - 1.12) p=0.025
Quintile 3	3,098 (42.1)	1.16 (1.10 – 1.23) p<0.001	1.12 (1.06 – 1.19) p<0.001	1,424 (9.3)	0.87 (0.81 – 0.92) p<0.001	1.03 (0.96 - 1.10) p=0.475
Quintile 4	2,060 (42.2)	1.17 (1.09 – 1.24) p<0.001	1.12 (1.05 – 1.20) p=0.001	1,102 (10.0)	0.94 (0.87 – 1.00) p=0.61	1.22 (1.13 – 1.32) p<0.001
Quintile 5 (least deprived)	649 (43.2)	1.21 (1.09 – 1.35) p<0.001	1.18 (1.05 – 1.33) p=0.005	447 (11.8)	1.13 (1.02 – 1.25) p-0.023	1.53 (1.36 – 1.72) p<0.001
Missing	488	-	-	1,015	-	-

a Numbers with missing data relate to all included individuals (n = 48,518 and 95,297).

b Missing tobacco consumption data defined as absence of recorded tobacco consumption units (e.g. cigarettes per day, grams of tobacco per week) in those with most recent smoking status recorded as “current smoker”.

c Inconsistent smoking status data defined as most recent smoking status recorded as “never smoker” plus previous documentation as either current or former smoker in primary care record.

### 3.2 Consistency of ‘never smoking’ status records

10.3 % (n = 9,826) of those invited had inconsistent smoking status data (both a most recent status of “never smoker” and a previous status of current or former smoker) in their primary care record. The proportion of records with smoking status inconsistencies varied widely between individual practices (range: 0.7% - 50.0%). The frequency of inconsistent data was lower among males than females (aOR:0.45; 0.43–0.47), higher among individuals from less deprived IMD quintiles (e.g., least vs most deprived quintile: aOR:1.53; 1.36–1.72), and higher across nearly all the ethnicity groups, especially those of Bangladeshi ethnicity (aOR:9.79; 9.10–10.57), when compared to white British groups (Table 1).

### 3.3 Concordance of primary care and self-reported data

For individuals who completed a telephone-based eligibility questionnaire (n = 29,698), self-reported smoking status (current, former or never) was concordant with individuals’ most recent primary care record in 75.3% of cases (Table 2). Higher odds of non-concordance were seen in those from the two least deprived IMD quintiles (vs most deprived), and lower odds among those last recorded as former smokers (aOR:0.80; 0.75–0.86) compared with current smokers. Increased time since smoking status was last updated was also associated with higher odds of non-concordance (vs those with last documented smoking status < 12 months previously) as was black Caribbean and “other” white ethnicity (relative to white British ethnicity).

**Table 2:** Frequency and independent predictors of discrepant smoking status responses between primary care and self-reported responses (all LHC invitation responders, n=29,698)

	All LHC invitation responders (n) 29,698	Non-concordant smoking status Records n (%) 7,338 (24.7%)	Unadjusted OR (95% CI)	Adjusted OR (95% CI)
Sex (missing = 1)				
Female	12,862	3,151 (24.5)	1.0	1.0
Male	16,835	4,187 (24.9)	1.02 (0.97 – 1.08) p=0.452	1.16 (1.09 – 1.23) p<0.001

Age groups (missing = 23)				
55-59	9,226	2,341 (25.4)	1.0	1.0
60-64	7,637	1,882 (24.6)	0.96 (0.90 – 1.03) p=0.276	0.97 (0.90 – 1.05) p=0.496
65-69	6,343	1,554 (24.5)	0.95 (0.89 – 1.03) p=0.216	0.94 (0.87 – 1.03) p=0.165
70-75	4,649	1,108 (23.8)	0.92 (0.85 – 1.00) p=0.47	0.88 (0.80 – 0.97) p=0.007
>75	1,820	451 (24.8)	0.97 (0.86 – 1.09) p=0.592	0.94 (0.82 – 1.07) p=0.357

Ethnicity (missing = 611)				
White				
British/mixed British	13,917	3,275 (23.5)	1.0	1.0
Irish	923	210 (22.8)	0.96 (0.82 – 1.12) p=0.588	0.99 (0.83 – 1.19) p=0.934
Other White	4,016	1,041 (25.9)	1.14 (1.05 – 1.23) p=0.002	1.13 (1.03 – 1.23) p=0.009

Asian or Asian British				
Indian or British Indian	1,250	322 (25.8)	1.13 (0.99 - 1.30) p=0.076	0.89 (0.77 – 1.03) p=0.128
Pakistani or British Pakistani	531	132 (24.9)	1.08 (0.99 – 1.31) p=0.480	0.90 (0.72 – 1.12) p=0.341
Bangladeshi or British Bangladeshi	1483	412 (27.8)	1.25 (1.11 0 1.41) p<0.001	0.98 (0.86 – 1.13) p=0.812
Other Asian	761	204 (26.8)	1.19 (1.01 – 1.40) p=0.039	1.10 (0.92 – 1.32) p=0.287

Black				
Caribbean	1223	338 (28.6)	1.24 (1.09 – 1.42) p=0.001	1.22 (1.06 – 1.41) p=0.007
African	736	197 (26.8)	1.19 (1.00 – 1.41) p=0.045	0.97 (0.81 – 1.17) p=0.764
Other black	523	132 (25.2)	1.1 (0.91 – 1.19) p=0.367	1.05 (0.85 – 1.32) p=0.638

Mixed				
White and black Caribbean	182	40 (22.0)	0.92 (0.65 – 1.30) p=0.623	0.84 (0.57 – 1.25) p=0.390
White and black African	82	19 (23.2)	0.98 (0.60 – 1.64) p=0.939	0.66 (0.37 – 1.20) p=0.170
White and Asian	118	28 (23.7)	1.01 (0.66 – 1.55) p=0.96	0.98 (0.62 – 1.56) p=0.938
Mixed - other	229	58 (25.3)	1.1 (0.82 - 1.49) p=0.526)	(0.74 – 1.45) p=0.856

Other				
Chinese	261	57 (21.8)	0.91 (0.68 – 1.22) p=0.523	0.81 (0.59 – 1.12) p=0.203
Other	1317	320 (24.3)	1.04 (0.91 – 1.19) p=0.532	0.97 (0.84 – 1.12) p=0.707
Not stated	460	126 (27.4)	1.23 (1.00 – 1.51) p=0.056	1.16 (0.92 – 1.46) p=0.207

Most recent smoking status (missing = 1)				
Current	11,859	2,382 (20.1)	1.0	1.0
Former	13,309	2,189 (16.4)	0.78 (0.74 – 0.84) p<0.001	0.80 (0.75 – 0.86) p<0.001
Never	3568	1,805 (50.6)	4.07 (3.76 – 4.41) p<0.001	4.14 (3.80 – 4.51) p<0.001
Unknown/other	960			

National Index of Multiple Deprivation (missing = 392)				
Quintile 1 (most deprived)	9,449	2,189 (23.2)	1.0	1.0
Quintile 2	8,601	2,052 (23.9)	1.04 (0.97 – 1.11) p=0.274	1.00 (0.93 – 1.08) p=0.996
Quintile 3	5,291	1,353 (25.6)	1.14 (1.05 – 1.23) p=0.001	1.09 (0.99 – 1.19) p=0.069
Quintile 4	4,361	1,195 (27.4)	1.25 (1.15 – 1.36) p<0.001	1.16 (1.05 – 1.27) p=0.003
Quintile 5 (least deprived)	1,604	464 (28.9)	1.35 (1.20 – 1.52) p<0.001	1.26 (1.10 – 1.45) p=0.001

Time since status last updated (missing = 24)				
<12 months	19,615	4,427 (22.6)	1.0	1.0
12–24 months	4,626	1,259 (27.2)	1.28 (1.19 – 1.39) p<0.001	1.21 (1.11 – 1.31) p<0.001
24–36 months	1,980	582 (29.4)	1.43 (1.29 – 1.58) p<0.001	1.29 (1.15 – 1.45) p<0.001
>36 months	3,453	1,067 (30.9)	1.53 (1.42 – 1.66) p<0.001	1.42 (1.29 – 1.56) p<0.001

Reported daily tobacco consumption varied significantly between primary care records and self-reported data, with a mean reported difference of 6.8 (95% CI: 6.18–7.18) fewer cigarettes per day reported in primary care records compared to self-reported telephone responses. Of those with both previous documentation of smoking and a most recent status of “never smoker”, 50.9% (n = 1,861) reported having smoked 100 cigarettes or more in their lifetime, and 11.8% (n = 433) were ultimately deemed eligible for LCS.

## 4 Discussion

We examined the completeness and validity of smoking history data from 251 primary care practices to identify individuals to invite for LCS eligibility assessment. Use of smoking status in addition to age reduced the number of individuals invited by over 70%, when compared to inviting by age criteria alone. The smoking status last recorded by primary care showed good concordance with self-reported telephone responses when this record was either current or former smoker, and in most cases had been updated within the past three years. However, half of those last recorded by primary care as "never smoker" but with previous documentation of smoking, self-reported a history of smoking during telephone risk-based eligibility assessment and a significant minority proved eligible for LCS. Across all measures of data quality, disparities by sociodemographic factors were identified, most notably ethnicity and deprivation.

## 5 Conclusions

Our findings suggest sufficient accuracy to support the use of “ever smoker” status in primary care records as a means of identifying individuals to invite for further lung cancer risk assessment and potential LDCT LCS. However, we would caution against relying solely on the most recently recorded instance of smoking status, particularly if this record is “never smoker”, as our findings demonstrate inconsistencies within the data which could wrongly preclude individuals from invitation. Our findings also suggest that primary care risk stratification for LCS beyond age and smoking status would be limited by data completeness and recency for more detailed parameters of smoking history, necessitating provision within LCS programmes for detailed eligibility assessment at an individual level. Further work is needed to identify those with no smoking data in primary care records and to understand factors influencing the described disparities in data accuracy across sociodemographic groups, to ensure equity in LCS invitation.

## 6 Contributions

The described protocol utilising primary care records to target LHC invitations was developed by the study management team for the SUMMIT Study, led by SMJ. JLD and HH prepared the manuscript for review and completed the data analysis. All authors contributed to the development of the manuscript and approved the final version.

## CRediT authorship contribution statement

**Jennifer L. Dickson:** Conceptualization, Methodology, Formal analysis, Investigation, Writing – original draft, Writing – review & editing, Visualization. **Helen Hall:** Formal analysis, Writing – original draft, Writing – review & editing, Visualization. **Carolyn Horst:** Writing – review & editing. **Sophie Tisi:** Writing – review & editing. **Priyam Verghese:** Writing – review & editing. **Sarah Worboys:** Writing – review & editing. **Andrew Perugia:** Software. **James Rusius:** Software. **Anne-Marie Mullin:** Writing – review & editing. **Jonathan Teague:** Writing – review & editing. **Laura Farrelly:** Writing – review & editing. **Vicky Bowyer:** Investigation, Writing – review & editing. **Kylie Gyertson:** Investigation, Writing – review & editing. **Fanta Bojang:** Writing – review & editing. **Claire Levermore:** Writing – review & editing. **Tania Anastasiadis:** Writing – review & editing. **John McCabe:** Data curation. **Anand Devaraj:** Writing – review & editing. **Arjun Nair:** Investigation, Writing – review & editing. **Neal Navani:** Conceptualization, Writing – review & editing. **Allan Hackshaw:** Validation, Writing – review & editing. **Samantha L. Quaife:** Conceptualization, Writing – original draft, Writing – review & editing, Visualization, Supervision. **Sam M. Janes:** Conceptualization, Writing – review & editing, Supervision.

## Declaration of Competing Interest

The authors declare that they have no known competing financial interests or personal relationships that could have appeared to influence the work reported in this paper.
